# Just translation? A socioecological justice lens on EU environmental governance and urban mobility transitions

**DOI:** 10.1007/s41358-022-00335-1

**Published:** 2022-12-01

**Authors:** Nils Stockmann, Antonia Graf

**Affiliations:** Institute of Political Science, University of Münster, Scharnhorststraße 100, 48151 Münster, Germany

**Keywords:** EU governance, Norm research, Mobility, Environmental policy, Environmental justice, EU Governance, Normenforschung, Mobilität, Umweltpolitik, Umweltgerechtigkeit

## Abstract

EU environmental policies such as the Ambient Air Quality Directive 2008/50 are highly relevant in this age of the looming climate crisis and interconnected sustainable transitions. However, implementation efforts such as low-emission zones, road pricing, and driving bans affect citizens in heterogenous situations and in ways that evoke questions of socioecological justice. This has resulted in an increasingly polarized reluctance to respective governance across Europe. The EU policy implementation literature often omits these less clearly operationalized norms that EU policies transport and pays little attention to how stakeholders in cities discursively and practically translate EU directives. Constructivist norm research underlines the importance of ‘localizing’ by highlighting that justice does matter for norm translation. The environmental justice concept has, however, not been systematically introduced and referenced in the norm research literature. This article offers a heuristic to address this research gap by combining a translation perspective from International Relations norm research with an environmental justice lens. Following the journey of the Air Quality Directive 2008/50, we ask how urban implementation configures the Directive’s environmental justice dimension and why this is important for effective and sustainable EU governance. Empirically, we focus on action plans and participation processes regarding Directive 2008/50 in Brussels, Glasgow, and Hamburg. As a result, we show that EU environmental governance unfolds at the local level as a dynamic contestation of different distributive justice claims that then translate into concrete policies. The analysis indicates that those policies must procedurally integrate local knowledge and identity formation to enable comprehensively just sustainable transformations.

## Abstract (de)

EU-Umweltnormen wie die Luftreinhalterichtlinie 2008/50 spielen eine herausragende Rolle in Zeiten der Klimakrise und damit verbundener Nachhaltigkeitstransformationen. Gleichzeitig betrifft die Implementation von Maßnahmen wie Luftreinhaltezonen, Straßennutzungsgebühren oder Fahrverboten unterschiedlich situierte Bürger*innen und zieht daher Fragen nach sozialökologischer Gerechtigkeit nach sich. Dies hat über Europa hinweg zu einer zurückhaltenden und zunehmend polarisierten Haltung gegenüber entsprechenden Governancemechanismen geführt. Die EU-Implementationsliteratur nimmt diese weniger klar operationalisierten Normen oft nicht in den Blick und misst der lokalen Übersetzung von EU-Richtlinien durch Stakeholder*innen nur nachgeordnete Bedeutung bei. Die konstruktivistische Normenforschung hingegen misst der Lokalisierung von Gerechtigkeitskonzeptionen unter dem Begriff der Normübersetzung höhere Bedeutung zu. Aspekte der Umweltgerechtigkeit sind in diesem Forschungsfeld allerdings bislang kaum systematisch aufgegriffen worden. Dieser Beitrag nimmt sich diesen Forschungslücken an und entwickelt einen heuristischen Rahmen, der das Konzept der Normübersetzung aus den Internationalen Beziehungen mit einer Umweltgerechtigkeitsperspektive verbindet. Wir folgen der Reise der Luftreinhalterichtlinie 2008/50 und fragen wie urbane Übersetzungen die Umweltgerechtigkeitsdimension der Richtlinie konfiguriert und warum dies bedeutsam für Fragen der effektiven und nachhaltigen EU-Governance generell ist. Empirisch fokussieren wir auf Implementationsstrategien und Beteiligungsprozesse hinsichtlich der Richtlinie 2008/50 in Brüssel, Glasgow und Hamburg. Wir zeigen das sich EU-Umweltgovernance in lokalen Räumen als Dynamik umstrittener Gerechtigkeitsansprüche und deren Übersetzung in politische Strategien und Maßnahmen entfaltet. Die Analyse verweist dabei auf den Bedarf der Integration von lokalisiertem Wissen und Identitäten zum Zweck ganzheitlich gerechter nachhaltiger Transformationen.

## Introduction

A consistent and effective transition of energy systems in Europe and beyond will not be achieved without a sustainable turnaround in the sector of transport & mobility (Sovacool et al. 2017; Agora Verkehrswende 2017). As commonplace as this assessment is in both academia and practice, research revealing the complex dynamics of sustainable mobility and energy transitions is still scarce. In particular, the sociocultural aspects of this fundamental transformation have only recently started to inform research agendas. These contributions question the technocentric view of mobility transitions and fortify more holistic view including sociocultural governance dynamics (Sonnberger and Graf 2021; Mullen and Marsden 2016; Schwanen et al. 2011).

Furthermore, mobility and energy governance are not clear-cut policy fields. Policies like the European Union Ambient Air Quality (AAQ) Directive 2008/50/EC neccesitate the transition of energy systems. At the same time, they stimulate national policymaking by setting air quality objectives for member states that are designed to prevent and combat air pollution. Thus, while AAQ regulation has most commonly been described as an issue for *EU environmental policy* (Gollata and Newig 2017; Lenschow et al. 2017), within the member states’ multilevel governance systems, cities face the highest burden from both road traffic and externalities such as air pollution (European Commission 2017; Banister 2008). Consequently, the diffusion of AAQ into national policies infiltrates local policies and thus becomes an urban issue (Bondarouk and Liefferink 2017). For example, in the form of driving bans and low emission zones, AAQ governance directly affects the everyday life of citizens and shapes their daily habits (Leger 2019; Cass and Faulconbridge 2017).

As part of a sustainable transition, the normative aim of ‘clean air’ is subject to political negotiation and conflict, not least because the goal of clean air is laden with aspects related to environmental justice norms which, inter alia, constitute the normative dimension of the AAQ directive. From this perspective, (clean) air must be regarded as a socially and politically constructed object of governance that evokes different understandings and discourses (Kurze and Lenschow 2018; McNamara 2017; Barbehön 2016) and is fundamentally contested (Wiener 2018). This sociocultural dimension of the AAQ norm is largely omitted in EU implementation research and is also insufficiently explored in constructivist norm research. Instead, we can readily see a high degree of technical operationalization regarding emission thresholds and other technical specifications within policies relating to AAQ. Questions about what exactly clean air *is*, which measures are appropriate and proportionate to use in order to achieve clean air, and who is responsible for (re-)installing and maintaining clean air, are all part of the debate but have not, thus far, addressed the (environmental) justice aspect. Not surprisingly, regulating AAQ often results in protests expressing perceived injustices, calls for the rejection of climate policies, and expressions of skepticism about authorities such as the EU (Arning and Ziefle 2020; Fraune and Knodt 2018). As a result, the sustainable transitions stipulated by AAQ policy feed into the growing awareness of the EU’s incapacity for hierarchical steering, especially in the environmental sector (Bondarouk and Mastenbroek 2018; Jordan and Lenschow 2010). Accordingly, successfully implementing norms presents itself as a practical quest and more than ‘just’ a self-sustaining diffusion process (Börzel and Risse 2012). This observation points to a further normative dimension of EU governance, specifically, that of socioecological justice norms which become productive within EU AAQ policy. Unlike the clear-cut operationalization of AAQ as an environmental problem, those justice norms are extremely complex and utterly ambiguous. Nevertheless, we argue they may not be omitted as part of a comprehensive analysis of the dynamics and effects of EU policymaking and governance (also see Mc Mahon 2018).

Based on these observations, we fortify norm-theoretical approach derived from International Relations (IR) with an environmental justice perspective that captures the local dynamics of EU AAQ governance. By considering EU governance dynamics as processes of *norm translation *(for an overview, see Draude 2017), we aim to grasp how complex and polysemic normative aspirations “travel” across different levels of EU policymaking as well as their “use” in urban contexts (Zwingel 2016, 2012). Hence, we argue that such a perspective enables a more comprehensive understanding that takes note of socio-spatial and socio-cultural dynamics of policymaking and governance within the EU. Advancing EU policy studies through this more critical approach is a worthwhile task as it enables us to access the interactive nature of local engagement with the socioecological justice components of EU policies, moving past the predominantly technical operationalization of EU policies.

We empirically implement this novel framework through an exploratory analysis of the local translation of justice norms relating to the EU Ambient Air Quality Directive 2008/50. We chose three European cities, Brussels, Glasgow, and Hamburg in which we examined the local translation dynamics using an integrated qualitative-interpretative analysis of the air quality plans that were in place in 2018 and the documentation of the associated participation processes. In doing so, we were able to elaborate on the normative dynamics of EU governance with the help of a justice lens from the EU level to scites, not only through hierarchical steering but as an interactive process of norm translation.

We proceed as follows. In Sect. 2*,* we begin by examining the case of AAQ regulation within the EU up to Directive 2008/50. We outline the inherent normative dimension, underline the local and everyday relevance of the directive and point to conflicts in the implementation. We then show in Sect. 3 how far Directive 2008/50 constitutes a norm for transporting such understandings of justice in the sense that it brings about a norm translation. We, therefore, review the literature on policy diffusion within the EU and explain how our reintroduction of an IR norm theoretical concept plus an environmental justice perspective enriches the study of sectoral EU policy making. To this end, we combine the norm-theoretical perspective with literature with an environmental justice perspective in order to specify the normative contents. Section 4 outlines our methodological approach and justifies our selection of Brussels, Glasgow, and Hamburg for our study. Section 5 presents the results of our analysis of the three cases in three stages. By *zooming in* on the three cases, we demonstrate how the AAQ directive has been translated into policies. *Looking through* a socioecological justice lens, we then re-read the norm translations taking place in the three case studies. Discussing our findings in light of the context factors for norm translation, such as overarching governance structures and discourses, we then *zoom out* again and derive more general findings about norm translation in EU governance. We wrap up our contribution by discussing the implications of our findings for the study and practice of EU governance and the limitations of our research in Sect. 6.

## Ambient air quality regulation in the EU

Globally, the issue of AAQ^1^ was acknowledged as a political concern in the 1970s when international organizations such as the World Health Organization (WHO) pointed to the damaging externalities caused by air pollution (Williams 2004). Therefore, we consider AAQ and the problems associated with it to be part of a global environmental governance architecture that covers a broad array of discourses and normative developments (Biermann et al. 2009). Despite the well-established relevance of AAQ within this architecture, the EU did not put forward a coherent strategy and legislation addressing air pollution and thereby ‘translating’ the global discourse into a standalone, workable *policy norm* until 25 years ago. The externalities of air pollution predominantly occur on a local scale, especially in cities affected by phenomena such as smog. Hence, the regulation of the problem was long deemed subject to the subsidiarity principle without substantial effects in implementation (Smeds and Cavoli 2021; van Kersbergen and Verbeek 2007). For a long time, the EU’s coordinated action was not perceived as “adding value” to national and local policies nor as monitoring national compliance on a larger scale.

This changed with the growing interest in the place of the “urban environment” within the emerging sustainable development agenda (Bulkeley and Betsill 2005). The 1992 Rio Conference placed the “quality of life” within cities on the EU’s political agenda. Livable cities, in this regard, were understood as contributing to the European aims of generating market integration and economic growth alongside social cohesion and sustainable development. As widespread air pollution was identified as a significant obstacle to that ideal across the majority of European cities, AAQ became a priority for EU political action in the environment portfolio (Selin and VanDeveer 2003).

The *Council Directive 96/62/EC *of 1996 marked the EU’s first comprehensive legal take on the matter. While setting out local thresholds for certain air pollutants for the first time, this directive was limited in scope—and success (Dossi 2017). Further legislation and strategy, each covering a relatively narrow aspect of AAQ policy, did emerge but were never coherently integrated. Nevertheless, AAQ continued to be a salient topic within EU environmental policy, as cities and member states were unable to meet the highly specific thresholds the Commission had defined. In response, the Commission sought redress for the legal infringement of the numerous breaches (Gollata and Newig 2017; Barbehön 2016).

Therefore, from 2001 on, the EU commission committed itself to a *Clean Air for Europe *(CAFE) process with the aim of establishing “[…] a system for developing, collecting and validating scientific information on the effects of air pollution” and supporting “effective implementation of the existing legislation on air quality and the eventual development of new proposals to favour the appropriate observance of the requisite measures” (Dossi 2017, p. 107). In 2005, the CAFE process led to the Commission’s 2005 *Thematic Strategy on Air Pollution *(COM 2005/446), which paved the way for the directive *2008/50/EC: On Ambient Air Quality and Cleaner Air for Europe *of 2008 which, in turn, aims to comprehensively integrate the different AAQ legislation within the EU up to that point.

Within the Directive and beyond, air pollution is understood as a local problem, which is why the member states are to designate “zones and agglomerations within which air quality is to be assessed and managed” (2008/50: Chapter I, Art. 4). One part of the Directive, namely the uniform measurement and documentation of emissions, requires immediate action by the member states or the delegated sub-state levels in these air quality zones. Meanwhile, the second central aspect of the Directive is reactive. Only the violation of the pollutant limit values set in the Directive requires the establishment of *air quality plans* (ibid.: Chapter IV, Art. 23) and *short-term action plans* (ibid.: Art. 24) for those specific regional/local areas.

Compliance with Directive 2008/50 proved to be a considerable challenge within virtually all EU member states after 2010 (Cancik 2011). Although air quality plans were implemented in many regions and cities, outlining measures to comply with the AAQ thresholds, in many cases, the air quality plans did not improve the performance regarding AAQ in the respective area, and, thus, had to be revised. In response, the EU Commission opened infringement procedures against various member states (European Commission 2018). Accordingly, member states and regional and local authorities were confronted with the need to implement more effective policies in response to Directive 2008/50. Regarding urban mobility, whether regulatory policies, such as (temporary) driving limitations and stricter vehicle standards are necessary for compliance with the EU regulation was debated with increasing frequency and intensity.^2^

The EU Directive 2008/50 puts forward an inherently justice-related AAQ governance. Right at the beginning of the explanatory memorandum, Directive 2008/50 references the 6th *Environmental Action Programme* (EAP) of the European Community from 2002, which states that there is a:

“[…] need to reduce pollution to levels which minimise harmful effects on human health, paying particular attention to sensitive populations, and the environment as a whole, to improve the monitoring and assessment of air quality including the deposition of pollutants and to provide information to the public.” (2008/50: paragraph 1)

With this conception of the issues surrounding AAQ, the 2002 EAP encapsulates the EU’s reason for becoming active in the field of AAQ policy, that is, to preserve or reinstate a normatively desirable state that promotes livable conditions for citizens and the preservation of the natural environment. At the same time, the Directive differentiates between different levels of affectedness and inclusiveness in the context of AAQ governance.^3^ This normative dimension of AAQ governance has also unfolded through the growing politicization of the issue, politization especially in urban contexts, where air pollution is being taken up as an issue by civil society movements and measure-related protestors (see, for instance, Loopmans et al. 2022).

The sustainable transformation, which the AAQ Directive 2008/50 is supposed to facilitate, often incites protests amidst the overall high acceptance of a necessary sustainable transition (Arning and Ziefle 2020). Protests unfold—of course not exclusively—in larger-scale discursive contexts. For example, citizens’ voices have been calling for accessibility and more general “freedom to drive a car” (Haas 2020; Sonnberger and Leger 2020). Some protests in transformation processes must also be perceived in the light of populist backsliding and post-truth politics (for instance, Fraune and Knodt 2018).^4^ For example, the “diesel scandal” of 2015 uncovered an industry-wide manipulation scheme regarding air pollutant emissions that raised the question of responsibility to the agenda of courts, policymakers, and the wider public alike (Gross and Sonnberger 2020; Palmer 2019). In France, the planned introduction of a new fuel tax, framed an attempt to internalize energy consumption costs, led to the 2019 *Gillet Jaunes* (“Yellow Vest”) protests. While attracting a wide range of supporters, the movement formed primarily around the claim that energy and mobility poverty represented an injustice nurtured through the proposed tax (Martiskainen et al. 2021).

Sustainable transitions, such those related to mobility, also have social offset and induce inherent dissatisfaction that continue to be voiced through protests especially in local contexts (Lucas 2012). Some prominent examples include the lawsuits the German NGO, the *Deutsche Umwelthilfe*, filed against various regional and local authorities following the introduction of driving bans in several German cities. For instance, the driving bans in Stuttgart incited harsh protests at the local level (Arning and Ziefle 2020; Sonnberger and Leger 2020). Although the dimension of protest and participation is much more multilayered and complex than we can show here, the examples provided demonstrated how important the justice dimension is when normative content travels. Studies of sectoral governance should therefore engage more extensively with these dynamics. Even though the interplay between *ideational components* and EU governance structures has already received some consideration within the discipline of EU studies (Borrás and Radaelli 2011; Skogstad and Schmidt 2011), these contributions have not, to the best of our knowledge, systematically referenced the local and sociocultural conflicts in norm dynamics and policy diffusion.

In the next section, we will explain this observation in further detail and, by combining a translation perspective from International Relations norm research with an environmental justice lens, offer a framework for addressing this research gap.

## EU policymaking as norm translation: integrating environmental justice

To better understand the prominent normative dimension of the EU’s AAQ regulations and their impact on local and everyday contexts, we now present a short overview of EU policy studies research on environmental governance in the multilevel system and highlight the work that still needs to be done. Second, we offer norm translation as an analytical tool to comprehensively address normative tension and contestation in the implementation dynamics of EU policymaking. Third and finally, we then combine norm translation with research on environmental justice to develop a sound framework to access the AAQ Directive 2008/50.

### Implementation & compliance in EU (environmental) governance

Given the necessity of local and regional implementation, the EU’s AAQ regulation is often considered a quest for multilevel governance (MLG) (Gollata and Newig 2017; Lenschow et al. 2017). In this vein, the process of implementing EU standards in the field of environmental policy cannot be described as a one-sided or straightforward process. Instead, successful implementation of environmental regulation depends on various factors at different political levels and involves other actors beyond governments. These various elements influence each other and, thus, modify their impact on the environment (Bondarouk et al. 2020; Fink and Ruffing 2017).

As EU studies have evolved, *compliance *with EU-level governance outputs has risen to become the dominant dependent variable of interest (Treib 2014). However, by approaching the issues from the perspective of compliance, EU studies prominently focuses on the outputs of implementation processes. It seldomly considers how actors actually put the normatively contested and ambiguous meaning of EU directives into action (two notable exceptions are Barbehön 2016; Paul 2012). The interactive dynamics or ‘translation’ of policies are absorbed into the analysis of the linear implementation processes of each EU member state, processes which often remain on the level of legal adaption. In other words, policy research mainly asks why actors do or do not follow and adopt policies put forward by the EU. Herewith, EU studies has sustained an implicit hierarchical bias that constitutes a reverberating impression of a one-way diffusion of EU policies over different levels of governance (Börzel and Risse 2012).

As a result of this development, many EU researchers have already pointed to incomplete policy diffusion processes within the EU that are not capable of providing for compliance with and implementation of EU policies in specific contexts (for the environmental sector see, for instance, Börzel and Buzogány 2019; Bondarouk and Mastenbroek 2018). Hence, the crisis invites a reconsideration of the long-established certainties of EU studies. This debate about the re-conceptualization of basic concepts, such as compliance, is happening right in the midst of mainstream EU studies (Heidbreder 2017; Thomann and Sager 2017). Nevertheless, the concept of crisis, as the new *status quo *of EU research, has also re-invited a broad scope of critical and interpretive approaches that focus on the relevance of discourses, power, and knowledge within EU governance (Bigo et al. 2021; Manners and Whitman 2016).^5^

### Norm translation as a critical-constructivist lens on EU governance

Constructivist scholarship from International Relations (IR) provides one promising reservoir for such a critique. To be sure, constructivist perspectives are already well-established within EU studies (Christiansen et al. 1999; Checkel and Moravcsik 2001). However, those contributions have tended to explore a ‘middle ground’ between rationalist and post-foundationalist research frameworks (Guzzini 2000) and more critically orientated researchers have pointed to their continuing disregard of language and identity (Epstein 2008), the disempowerment of actors (Krook and True 2012) and the disrespect of knowledge from the global south (Epstein 2012; Acharya 2014).

As critical observers of the field have highlighted (Manners and Whitman 2016), such perspectives are also of critical relevance for the study of EU policymaking and governance. Building upon this line of research, we assert that applying a *translation perspective* to the dynamics of the MLG system, such as that found in the EU, enables us to gain a better grasp on the interactive potentials and agency of actors across different levels of governance when implementing norms. It hereby strengthens the interdependencies rather than a sequence of MLG interactions as the ‘meaning’ of one level’s normative aspiration relies on the potential for translation on another level. Rather than reinforcing legal compliance, norm translation highlights the substantial dimension of norm implementation, that is, whether a normative problem is discursively taken up and translated “into use” (Wiener 2009). Therefore, it also allows us to grasp the interactive and dynamic contestation of EU regulation as it is locally implemented and adapted. We suggest that it is useful to conceive of norms as temporarily fixed discourses (Graf 2016) that obtain a prescriptive quality in the sense that they set out “standards of appropriate behavior for actors with a given identity” (Finnemore and Sikkink 1998, p. 981). As such, norms, when codified in law or a law-like manner, link a normatively defined problem grounded in values to a practice that is deemed a suitable resolution to the problem (Winston 2018). Put differently, norms function as “vehicles” (Graf et al. 2021) that transport normative meanings over political realms to where they can be unpacked and translated (Zwingel 2017, 2016).

Moving forward on this basis, we understand norm translation as the operationalization of a norm’s meaning in a specific technical, social, or geographic context (Zwingel 2016; Haglund und Aggarwal 2011). The concept, thereby, contributes to a critical-constructivist tradition of norm research in which norms are not stable objects that are diffused or socialized in a hierarchical, linear manner. Instead, they are, by default, contested and re-defined in interactive and circular dynamics (Linsenmaier et al. 2021; Zimmermann 2017b; Krook and True 2012). Thus, while actors may attempt to make a norm and its content as “robust” as possible (Deitelhoff and Zimmermann 2018), norm translation highlights the room for agency in norm dynamics countering the dichotomous understanding of norm senders/entrepreneurs and norm takers, respectively (Wiener 2017). Norms must therefore be understood as “meaning-in-use” (Wiener 2009) and, thus, effective only if they are translated into local policy discourses and practices (Zimmermann 2017a). *Hence, we understand norm translation as the travel, operationalization, and implementation of normative ‘meaning’ across different political and technical realms.*

As demonstrated, we can better understand the interactive dynamics that unfold in the local implementation of ideational components of EU policy, such as in AAQ regulation, through a norm-translation approach. We recognize the Directive as a vehicle to transport and operationalize normative aspirations for an *environmentally just *AAQ regulation. In particular, norm translation offers a starting point from which to consider the agency of ‘the local’ in setting ‘meaning-in-use’ concerning the governance of ‘problems’ that are inscribed into technical provisions such as thresholds or spatial boundaries. The approach therefore allows to extend the analytical focus beyond legal and technical implementation of norms and additionally sheds light on the justice-related and potentially ambiguous normative impetuses norms transport. Furthermore, the notion of norm translation implies that norms do not originate on a particular level of a given MLG system, just as the associated discourses are not confined to one level (Barbehön 2016). Nevertheless, the codification of norms does occur at a specific level, in the case of AAQ regulations, at the level of EU institutions. *Given this, we understand norms as provisions within EU policy documents that include an actor-guiding dimension.*

### Explicating environmental justice and norm translation

Turning a norm-theoretical lens onto the EU’s AAQ governance provides a more comprehensive focus on the implementation of the normative aspects relating to AAQ as such, e.g., the rationale for complying with air pollutant thresholds as defined by Directive 2008/50. Moreover, we propose that a norm translation perspective also allows for the analysis of more of the implicit meaning that is transported via the AAQ directive, particularly in relation to environmental justice norms. This, in our understanding, adds a further normative layer that has, until now, not received substantial attention in either EU policy or governance studies.

A more theoretically elaborate understanding may serve as an analytical vehicle for accessing how the social-theoretical concept of justice is addressed by (international) norms (see, for instance, Wiener 2018; Björkdahl 2002). In the language of norm research, actors ought to behave in a way that is appropriate to the production of a just society. However, *what is just *in any particular context at any particular time is contested academically and practically. Therefore, some further explanation is required in order to clarify which particular understanding of justice informs our analysis.

Scholarship on *environmental justice* has long been pointing to the intersections between the ecological and social dimensions of sustainability-related policies and transitional processes (Dobson 2010; Stephens 2007; Agyeman und Evans 2004). From the conceptual breadth of justice theories, the critical and communitarian approaches in particular have provided analytical tools for grappling with the structural complexity at this intersection (Schlosberg 2013).^6^ As a result, environmental justice is now commonly understood as a multidimensional concept (Walker 2012; Schlosberg 2007). Moreover, processes and conflicts regarding environmental justice, though still related while relating to broader discourses, are recognized as, first and foremost, accessible in local particularities. Here, urbanized contexts in particular have already received vast attention in the literature (Moroni 2020; Gould und Lewis 2017).

Air pollution, like environmental pollution in general, has been a central issue in the environmental justice literature from the very beginning of the scholarly debate (Agyeman et al. 2016; McGurty 1997). It has also been substantively described as a locally grounded phenomenon that is highly relevant to managing climate change, especially within urban contexts (Graham 2015). Therefore, we consider it another topic that is well established within the environmental justice literature that is particularly amenable to translation across political realms via policies such as the EU Directive 2008/50.

Air pollution is not evenly distributed but concentrated at the sites where pollutants are emitted. Therefore, people living or working at or close to such sites are more affected by the pollution and face a higher risk of experiencing negative consequences (Buzzelli 2020). As many studies have pointed out, this constitutes an environmental injustice when specific social groups belonging, for example, to a specific race, class, or gender, are overrepresented in these areas of greater air pollution (Mitchell and Dorling 2016; Jerrett 2009). Research has also highlighted social justice questions arising from transitional processes in sectoral policies such as energy and mobility (Mattioli 2016; Mullen and Marsden 2016). This *distributive justice dimension* addresses the issue that particular demographic and social groups may have less access to the ‘good’ of a healthy life than others because they are more exposed to and affected by air pollution or because they are particularly affected by the measures instituted to combat air pollution and other environmental concerns. As one of the foremost perpetrators of air pollution, the mobility-energy nexus plays a focal role in addressing the injustices mentioned above. Within cities across all EU member states, road traffic, in particular, accounts for the majority of emissions and only slight reductions in emissions have been achieved to date (Loopmans et al. 2022; European Environmental Agency 2018). In line with the “Polluter Pay” principle at the core of environmental justice debates, it is apparent that the transport and mobility sector is expected to contribute to substantially improving AAQ.

However, in light of the social inequalities that have been identified, scholars and practitioners of environmental justice have argued that a *procedural dimension* of justice is needed to complement the distributive dimension (Gould and Lewis 2017; Schlosberg 2007). Procedural justice starts from the assumption that injustices need to be voiced and heard in political and social processes if they are to be acknowledged and integrated into policy solutions. It aims to make accessible the specific knowledge of vulnerable and excluded groups and identify intersectional dynamics and responsibilities for structurally embedded injustices (Ottinger 2013; Lidskog and Sundqvist 2011). Combined with the broader understanding of a recognition dimension of justice, these dimensions constitute what Walker (2012) has defined as a “tripartite structure of environmental justice”.

Walker’s (2012) approach of *claim-making* links the different justice dimensions and provides a heuristic to further operationalize environmental justice as a norm that is translated through EU AAQ policy. In this vein distributive and procedural justice components can be understood as objects that describe “how things ought to be” (Walker 2012, p. 40) and, thus, induce normative desirability. This, in turn, corresponds well with the norm-theoretical understanding we established earlier. However, as Walker argues, when reality ‘is’ different from the desired state *(evidence)*, this can be explained by *processes *that determine “why things are how they are” (ibid.). It then follows that altering these processes to realize the desirable state requires behavioral change that can be induced through *norms*. However, justice claims, as understood by Walker, are contested by default. In the previous section, we also underscored this impression by pointing to the protests and opposition to AAQ policies. These articulations also incorporate claims about injustices and negotiating these clusters of claims requires a political process in which the various claims are articulated and justified. As a result, the norm is unpacked and translated into locally accessible aspects of justice, evidence, and processes that may potentially come into conflict and collide. In this vein, Walker’s perspective helps us make the justice norms transported through AAQ policies visible and accessible.

As a result of this engagement with the environmental justice perspective, we find that the study of AAQ governance must not stop at assessing the (legal) compliance with thresholds and further technically specified norms as put forward inter alia by EU policies. Rather, it is also important to consider the additional normative content of AAQ regulation such as justice norms in sustainable transitions such as that of mobility and transport. While these norms often follow a less precise, that is, a more ambiguous norm translation approach, they can, we suggest, serve as an analytical heuristic for discovering the dynamics of justice-related meaning within multilevel AAQ governance. This implies a structurally and discursively politicized character of norm implementation processes. Amid seemingly clear technical operationalizations, the translation of AAQ policy potentially fuels conflicts in the political order that manifest themselves in specific localities.

Taking into account a multidimensional understanding of environmental justice and understanding local particularities as single cases that have to be accessed separately, we propose structuring the analysis around three main, interrelated categories (see Table 1). Regarding the dimension of distributive justice, we investigated claims that were reiterated in local translations of the EU AAQ policy, both, relating to the effects of air pollution as well as the effects of measures instituted to combat air pollution. We suggest there is a normative tension arising in this dimension. The category of procedural justice covers the preconditions for making such claims visible within the local discourse about AAQ governance. We suggest that mechanisms for the inclusion and exclusion of actors and knowledge become apparent in the analysis of local cases and that this signifies different levels of access to claim-making. Finally, we assume that factors relating to the governance structure of local contexts as well as extant discourses play a contextual role in understanding the respective translation dynamics. While we do not suppose a causal interference for these factors, we still consider it necessary to account for them in a comprehensive analysis of norm translation dynamics.

**Table 1:** Categories and guiding questions for the analysis

**Case assessment**
*What (regulatory) measures regarding mobility were introduced in response to the AAQ Directive 2008/50?*
**Category 1: Distributive Justice**
*How is the inventory of AAQ Directive 2008/50 translated from a distributive socioecological justice perspective?*
**Category 2: Procedural Justice**
*How is the inventory of AAQ Directive 2008/50 translated from a procedural socioecological justice perspective (including questions of recognition)?*
**Category 3: Contextual Factors**
*What particular governance structures and extant discourses affect the norm translations in the context of the AAQ Directive 2008/50?*

## Methods

In this contribution, we qualitatively analyze the dynamics of local mobility-energy transitions that are key to translating the EU’s AAQ Directive 2008/50 and its inherent justice dimensions in three case studies: Brussels, Glasgow, and Hamburg. When a translation of the socioecological justice norms occurs, we expect to find discursive and practical manifestations of this norm within our corpus, as well as the measures they envisage, and the process that has been written and discussed. In this section, we will elaborate further on the selection of the three cases and then clarify each individual step of the analysis.

### Case selection

Ambient air pollution continues to be a challenge for many cities in practically all EU member states (European Environmental Agency 2020). Even though there have been recent improvements in many places—not least during the course of the Covid-19 pandemic (Zehl and Weber 2020)—there remained a large number of possible cases for this study. In fact, in 2015, the European Environmental Agency (EEA) identified 156 cities in 21 EU member states that breached nitrogen oxide thresholds (European Environmental Agency 2018). From this ‘population’, we chose three cases, Brussels, Glasgow, and Hamburg, for an in-depth, comparative analysis.

While Berking and Frank (2010, p. 168; own translation) rightfully conclude that “there is something arbitrary” about any city-specific case selection, in selecting our sample, we did consider aspects beyond pragmatic factors:

- A superficial (socio)geographical examination of the selected cases showed that they are similar in various ways. As important metropolitan regions, they are regionally exposed within their national contexts and are thus an important reference point for mobility and economy. This is reflected, for example, in that they placed great importance on (inner-)urban transport, especially for commuters (European Commission 2017). The pollution situation due to air pollutants in 2018 was also significantly above the EU average in all three cases (ibid.; European Environmental Agency 2018). Consequently, all three selected cases have been pursuing systematic AAQ planning since the beginning of the 2000s.
- An explorative first examination also revealed the presence of different challenges and structures that made an evaluationof the individual cases appear interesting. Most importantly all three cases examined are located in EU member states with a high degree of decentralization. In addition to the relevant local actors, we were able to identify key actors on other various political levels that enabled a multilevel understanding. Brussels and Hamburg, in particular, both have notable statuses: Brussels governs the Brussels-Capital Region within the Belgian federal structure, while Hamburg has an extraordinary role as a separate federal state in Germany. Glasgow also has a unique feature in that many of the regulations regarding AAQ policies are influenced or even directly legally implemented by the British central government in London. Furthermore, Glasgow has a significant influence over the Scottish regional government.

Evidently, these criteria do not sustain a thorough comparative analysis of the selected cases, which is also not the aim of this article. Rather, we conceive of the three cases as standalone examples for the translation of EU norms among different realms of governance that facilitate an exploratory analysis that is primarily focused on uncovering the dynamics related to the conceptual puzzle outlined above. However, we will come back to such particulars in our conclusion.

As highlighted before, the transport and mobility sector is generally attributed a focal role within the implementation and compliance strategies relating to the regulation of AAQ. Naturally, local strategies to combat air pollution are more comprehensive and, indeed, we must suppose that justice norms also play a significant role in sectors such as energy and housing (see Jenkins et al. 2016; Kohlhuber et al. 2006). Nonetheless, in our analysis, we focus on transport and mobility because of the apparently high salience and politicization of justice claims related to localized air quality strategies within this particular sector as compared to other sectors included in the local air quality strategies.

### Methodical approach

We analyzed selected cases, building on a hermeneutic content analysis of the compliance of local strategies with the EU Air Quality Directive 2008/50. In particular, the 2018 *air quality plans* for the individual cities, as well as the *supplementary declarations and documentation* formed the primary material for analysis. Thus, the focus was on concrete strategies that translated the EU Directive to the respective urban contexts. However, this material had to be supplemented in order to trace the discursive level of norm translation. For this purpose, the *documentation of local participation processes and political discussions* provided a good starting point.^7^

As with the framework legislation of the EU, the norm dynamics accessed within the three cases must be understood in the context of the national legislation that formally instates EU Directive 2008/50 into law. These national legal acts^8^ are, of course, important for our analysis, as they set out the legal national reference point (top-down in relation to European Commission infringements, as well as bottom-up through public/private litigations) for the implementation and proportionality of the concrete measures introduced in the three respective cases. Nonetheless, as we observed that claim-making related to the justice dimension of the Directive happens primarily in local contexts, we did not consider the national transpositions in this formal analysis.

For the analysis of local documents, we used the two dimensions identified in the analytical framework as deductive categories, distributive and procedural justice, which we then supplemented with a third category of possible contextual factors within the governance system and extant discourses. To structure our analysis, we applied the guiding questions derived in the previous chapter to each category.

We applied the categories to the selected material in the analysis using the qualitative content analysis method proposed by Kuckartz (2010). Thus, in an inductive procedure, we identified sub-codes referring to these three categories within the material to account for the local translation of the justice dimension of the EU AQQ Directive. We also left space to identify and interpret other relevant material, such as media coverage or statements by social actors and findings complementing the category systems, which were not included in the content analysis. For this process, we utilized the software Maxqda, which allowed us to code a comparatively large corpus of texts for each of the cases with a multilingual, lexical keyword search. We then coded the text passages identified within the documents manually, which allowed our review of the lexical search and the keyword system to be expanded inductively (cf. Kuckartz 2010). Again, following Kuckartz’s concept, the coded text passages were then generalized and reduced in several stages.

## Results

Our in-depth analysis of three norm translations of the EU AAQ Directive 2008/50 in Brussels, Hamburg, and Glasgow showed how informal structures and dynamics influence the scope and characteristics of norm translation. This section presents the results in more detail as if through a microscope (taking inspiration from Nicolini 2009). We first *zoom in* on the specific measures deployed in each case to translate the EU norm. Then, we provide more detailed descriptions of the regulatory measures that we anticipated triggered justice claims. Secondly, we *look through* the justice lens to pay closer attention to the meaning of the justice-related content that unfolds in each case focusing on the distributional and procedural justice dimensions (*Categories 1 & 2*). Thirdly, we *zoom out* from the individual cases to discuss the learnings about norm translation processes we can derive from our analysis. This step refers primarily to the political-structural and discursive context factors (*Category 3*) that intervene in the local governance of air quality.

### Zooming in: translating the EU’s AAQ directive 2008/50 into local contexts

As our initial exploration of the available material in the three case studies showed, compliance with the AAQ Directive 2008/50 was perceptibly problematic in all three cities considered, as was demonstrated by the media and political attention they received. Furthermore, all three cities updated their respective air quality plans between 2016 and 2017 in response to the public awareness of the issue and the political pressure calling for them to address their persistent non-compliance with the Directive. These observations relate to the technically framed normative impetus of the Directive of (a) providing an operationalization of a problem (air pollution tresholds), (b) the measurement thereof, and (c) a procedural reaction, here, the establishment and revision of air quality plans.

In all three cases, regulatory interventions in the transport and mobility sector represented the anchor points of the air quality plans. By introducing physical/legal barriers or imposing sanctions, regulatory measures in the context of (urban) mobility alter the answer to the question “How/where can I move with what means of transport?” and are thus intervening in the *status quo *of mobility practices (Sonnberger et al. 2019). As we understand, in particular, these kinds of measures as a trigger for protests involving justice claims, we here limit our discussion to those elements.

In response to the AAQ Directive 2008/50, the City of Hamburg introduced driving bans for diesel vehicles in two streets particularly affected by air pollution. In Brussels, a comprehensive low-emission zone was introduced at the beginning of 2018 which stipulated that vehicles of certain pollutant classes were no longer allowed to enter the area. In the case of critical threshold violations in the zone, all traffic was to be shut down. Finally, in Glasgow, a comprehensive low-emission zone was implemented step-by-step in 2018, replacing the previous zones that were restricted to individual streets. More thorough speed limits were also discussed as possible options in all three cases. Thus, measures beyond ‘soft’ incentive mechanisms were provided for within the design of the air quality plans. Consequently, we observed justice-related normative tensions related to all of the chosen cases. In all three cities, air pollution remained a persistent problem *and*, in all three cases, restrictive measures in the field of transport and mobility were implemented in an attempt to address this problem. Table 2 gives an overview of the most prominent measures discussed and/or implemented in each case and generalizes the effects of these policies at the urban level.

**Table 2:** Measures introduced in the air quality plans of the three cases

Measure	Effect
**Brussels**	
Low Emission Zone (*lage-emissiezone*)—implemented	Vehicles above a certain emission standard are prohibited from entering
Urban Access Fee (*péage*)—***not*** implemented	Cost internalization for vehicles entering the urban area
Driving bans in case of acute AAQ conditions—implemented	(Temporary) prohibition of entry for all private vehicles
**Glasgow**	
Low Emission Zone—implemented	Vehicles above a certain emission standard are prohibited from entering
Restrictive parking management—implemented	Reduced (or charged) parking areas
**Hamburg**	
Driving bans for diesel cars in certain streets in the urban area—implemented	Diesel cars above a certain emission standard are prohibited from entering
A general speed limit of 30 km/h—***not*** implemented	Speed limit reduced in the whole urban area
Restrictive parking management in certain areas—implemented	The number of parking areas is reduced

In all cases, a large number of incentive-based and educational measures complemented these regulatory interventions. As our analysis showed, those measures were often prioritized, whereas the regulatory interventions were framed as “last resorts”. Not surprisingly, we found several effects which showed the depth of engagement with daily routines and every-day-habits. Big trucks had to take detours, less inner-city space was available for parking, and older cars could not access specific areas. Apparently, in a suspenseful relationship the adaption of overarching technical and procedural provisions is amalgamated with a politicized translation situation in local realms. We witness emerging spaces in which different claims are articulated and conflict about different such interpretations are deliberated. This observation already points towards the (politically anticipated) justice effects of the regulatory measures that we consider in the next section.

### Seeing AAQ through a justice lens: findings on distributional and procedural justice

The potential justice-related effects of the measures outlined above must be addressed as a norm translation via the political deliberation about conflicting justice claims. If successfully voiced within this translation, justice claims translate into (soft) policy measures to mitigate the justice effects of the regulatory interventions. Approaching these dynamics, in Table 3, we summarize the inductively created code-sets for the first two categories that guided the analysis, namely the distributional and procedural aspects of justice, and that were addressed across the three cases. At this point, the inductive code sets (column 3) no longer represent specific measures introduced in the respective cases. Instead, we focus on the discursive uptake of claims and associated strategies designed to address potential injustices. This section will further examine which code sets were represented and which particular translation the respective cities demonstrated.

**Table 3:** Category system for the analysis & inductive code sets of the justice dimensions of the EU’s AAQ Directive 2008/50

Guiding questions (see also Sect. 3)	Inductive code sets	Anchor examples
**1: Distributive Justice**		
How is the inventory of AAQ Directive 2008/50 translated from a distributive socioecological justice perspective?	*1.1 *Unequal affectedness/risk from air pollution is recognized and addressed	Persons with medical preconditions, children, elderly (all cases), homeless people (Brussels)
	*1.2 *Strategies take into account the possible justice-related effects of the proposed measures	Effects on commercial traffic (Hamburg), mobility needs of elderly people, low-income households (Glasgow)
	*1.3 *Discussed measures are related to cost-internalization and compensation	Exemption rules, rejection of large-scale low-emission zone (Hamburg), free or reduced-fare public transport (Brussels)
	*1.4 *Socio-spatial aspects are recognized	Distribution of measurement stations, recognition of the spatial distribution of services
	*1.5 *Attribution of responsibility	Local companies (Brussels), federal level, car manufacturers (diesel scandal), evaluation
**2: Procedural Justice**		
How is the inventory of the AAQ Directive 2008/50 translated from a procedural socioecological justice perspective (including questions of recognition)?	*2.1 *Data collection is representative and comprehensive	Inclusion of complementary data sources, extension of categories/indicators, inclusion of (social-)scientific studies
	*2.2 *Participation opportunities in strategy development	Digital participation tools (Brussels)
	*2.3 *Accessibility of data and strategic documents	Use of understandable language, reprocessing of data/material, education strategies
	*2.4 *Integration of and with local knowledge	Outreach procedures in place, knowledge about the object of ‘clean air’, cooperative language
	*2.5 *Building partnerships/cooperations	Public-private partnerships with companies (Brussels), inclusion of local civil-society initiatives
	*2.6 *Identity/narrative formation	City inhabitants as ‘in-group’, local sustainability strategies, placemaking, (natural/cultural) heritage

#### The distributive justice dimension

Aspects of distributive and procedural justice were named and politically translated in all three cases considered. Particularly in the area of distributive justice issues (Category 1), a close relationship between the vulnerability of certain social groups and air pollution was identified in all cases, and this relationship was also present at the EU level. While children, the elderly, and people with medical preconditions were named in all cases, Brussels stood out as it also identified homeless people as being particularly affected. The importance of fulfilling this claim for justice was emphasized in all three contexts. Indeed, it was used as a justification for intervention in other areas, especially in the mobility sector. Measures to maintain or achieve a conveniently suitable AAQ are very similar in all three cases, for example, in increasing the attractiveness of alternative modes of transport to the car (so-called ‘push’ measures) or regulatory interventions such as transit/entry restrictions or parking space shortages (so-called ‘pull’ measures). However, the three cases differ considerably in terms of being a ‘just’ design. While in Hamburg, commercial traffic was identified as being significantly affected by injustices, the plan design in Glasgow focused more on the mobility needs of individual people. Once again, a central focus was placed on their social vulnerability:

“However, it is likely that in some cases local authorities will identify measures that could impact individuals and businesses—such as charging clean air zones—as the fastest means of achieving compliance. Depending on the scope of vehicles covered by such a zone this could particularly impact on low income families, small businesses and people living or working in a particular area.” (Consultation B, Glasgow: p. 5)

In contrast, companies in Brussels, despite being in a different position of responsibility (as will be explained later), are even more strongly obliged to contribute to the management of AAQ than in the other case cities. Even more than those affected by the injustices, businesses are seen here as part of the cause of it:

“The improvement of air quality and the health of the citizens of Brussels can only be effectively met by adopting measures that affect all car users in the region.” (LRP RBC: p. 42, own translation)

Accordingly, the measures thought to achieve distributive justice within the air quality management plan vary: Hamburg is considering numerous exemptions for personal and industrial traffic in its driving bans, while a large-scale low-emission zone has been categorically rejected. The concern that this approach could jeopardize compliance with the justice objective of AAQ, therefore, plays a smaller role in Hamburg than in the other two cases. Other justice-related compensations, such as the free carriage of bicycles on public transport or even a so-called “citizen’s ticket”, are also viewed rather suspiciously in Hamburg:

“Some expect a citizen’s ticket to lead to a sharp increase in passenger numbers. However, the possibilities (even in the medium term) of providing the necessary capacities for such a sharp increase would be limited.” (LRP FHH, p. 64, own translation)

In contrast to Hamburg, low-emission zones were established in both Brussels and Glasgow. In particular, the Brussels zone is characterized by *(a)* a high stringency in terms of enforcement (Brussels Environment 2021) and *(b)* high sensitivity to those potentially affected. The low-emission zone has been integrated into a holistic mobility concept (e.g., parking spaces close to the city with good public transport connections) and, in the case of complete driving bans in the event of acute pollution peaks, public transport is to be free of charge. In Glasgow, the adaptiveness of the low-emission zone seems less predictable—here, the plans for the zone as an instrument have been identified and prioritized by other political levels.

#### The procedural justice dimension

There are also significant differences between the translations of procedural justice aspects (Category 2) in the three urban contexts here considered. Although participatory procedures were carried out to shape and create acceptance of the air quality plans in all cases, we are here, qualitatively speaking, evaluating them differently. Hamburg’s air quality planning faced massive criticism from civil society because there was no interest in involving the city’s residents. Nobody has disputed that the responsible Senate administration complied with German and European law when they designed the procedure described above. However, existing leeway was not used in comparison to the other cases:

“Associations, interest groups, and, in particular, the citizens of the city of Hamburg who are affected when the limit values are exceeded should and could have been involved as early as possible in the preparation of the plan—e.g., within the framework of workshops or at least by setting up a citizen participation portal, as is common practice in other cities.” (LRP FHH Einwendungen: p. 10, own translation)

Based on the documents analyzed, the same applies to other aspects of procedural justice, such as representativeness and transparency. Here, too, the applicable regulations are being complied with, justifying non-action beyond the minimum “requirements” stipulated. In the two other cases considered, we also found similar references to the applicable laws. However, the analysis of the participation formats shows a higher sensitivity to the actual objections/proposals in Glasgow and Brussels. In this respect, as can be seen from the analysis of the documents, these two cities put more outstanding effort into approaching individual interest groups through targeted ‘outreach’ and considered their needs in their proposals. In both these cases, the empowerment of individuals through the dissemination of knowledge about air pollution and alternative forms of mobility (e.g., public education) is an essential prerequisite for a just translation of Directive 2008/50. Creative, often long-term solutions and education strategies complement the minimum requirements suggested by the EU Directive in many places.

The involvement of local civil society, economic actors, and individual subjects with their personal concerns is thus a central factor. The analysis of the documents for Hamburg reveals a kind of ‘front position’ between the administration as the author of the air quality plan, and other actors—as has already become apparent in the previous comparison. In particular, the sources documenting the direct confrontation give the impression of resistance to criticism of the air quality plan. Thus, although references to Hamburg’s identity include “the people who live and work there”, in some places, it seems as if this very identity is being defended against rather than together with the groups and individuals concerned. Furthermore, we identified contrasting narratives in the other two cases. Brussels’ air quality plan is embedded in a regional sustainability strategy that emphasizes the preservation of the city and its special position within Belgium is a central concern. At the same time, the inclusion of local initiatives, for example, in individual districts/neighborhoods, and the reference to socio-economic guidelines beyond those for air pollution control such as the ‘circular economy’ (or *économie circulaire*; RBC: p. 12), creates opportunities for sustainable inner-city dynamics. Similarly, although not as comprehensive and formative, the development of the air quality plan in Glasgow also sought to institute overall societal guidelines and identity features. Here, for example, the concept of “placemaking” (Report: p. 22; air quality plan GLA: p. 35) was used as a guideline for the idea of a sustainably changing urban space. The firm reference to the natural heritage of Scotland, beyond the city of Glasgow, is also formulated against this backdrop of the identity-shaping translation of the EU directive.

In all three cases, the role of science, knowledge, and technology is discussed, yet again, from different perspectives. Technological progress is framed as a prerequisite for the successful implementation of EU Directive 2008/50. Progress, in this sense, is only possible if reliable and affordable technology is available to the public sector, companies, and private individuals alike. Thus, it is identified as a necessary condition in all three cases, not least concerning the necessary integrity of the technology—especially in the results of a keyword search for ‘diesel scandal’. Technical progress as a level of action is, therefore, inevitably added to a necessary social identification and transformation. Yet, the cases differ in terms of who obtains the authority to generate knowledge about air pollution, its consequences, and effective ways of containing it. In Hamburg, as in Brussels, concepts that promote cooperation between private and civil actors in the development of better knowledge are opposed by references to EU or federal requirements that regulate the necessary ‘quality’ of knowledge. In contrast, in Glasgow and the United Kingdom, the justification for the plan itself refers to scientific studies beyond the publications of international actors such as the EU. Who has the authority to provide evidence for air pollution or the assessment of AAQ measures is a question that visibly influences the translation processes under consideration:

“Local community empowerment was seen as a useful tool in generating local engagement as this would create a platform for sharing information at a local level; encouraging citizens to participate in collecting air quality data.” (Consultation A: p. 9)

It is from this perspective, through a ‘justice lens’, that we observe that the regulatory measures to address persistent problems regarding AAQ (with an inherent distributive justice dimension) are moderated in the local discourse on implementation through justice claims referring to the (potential) effects of the mobility measures. In all three cases analyzed, these discourses translate into concrete policies that mitigated these just effects. However, this mitigation also raised the question of the effectiveness of the regulatory measures in terms of producing a ‘just’ state regarding the effects of air pollution. Therefore, the translation of justice remains a dynamic, contested equilibrium that unfolds in the political arena.

### Zooming out: discussing translation dynamics of EU norms

The various normatively-loaded meanings of justice found within the local AAQ policies can be specifically illuminated against the background of the path of norm translation by adding the contextual factors also examined (Category 3, Table 4).

**Table 4:** Contextual factors for the norm translation into urban policy

Guiding questions (see also Sect. 3)	Inductive code sets	Anchor examples
**3: Contextual Factors**		
What political-structural factors and extant discourses affect normative translation of the AAQ Directive 2008/50?	*3.1 *Reference to the competencies of other levels	Fragmentation of competencies, support of individual measures, resource dependency, feedback effects on other levels
	*3.2 *Legal interventions	Precautious behavior
	*3.3 *Discussions regarding resources and conflicts of interest	Resource scarcity
	*3.4 *Embedding regulation into comprehensive strategies/processes	Making regulation ‘a work on its own’

Even a cursory examination of the analyzed material shows a significantly different fragmentation of the three translations considered. In Hamburg and Brussels, the respective environmental authorities of the region were responsible for the final design of the translation. Meanwhile, in Glasgow, the picture of a strongly fragmented ‘translator community’ emerged. In addition to the local level, the regional level (of Scotland) and the national level were also significantly involved in shaping the plan. As, according to the document analysis, there are apparent conflicts between these levels concerning the distribution of responsibility and resources, for example, in support of local environmental zones, the impact of the dimensions of environmental justice is also potentially contributing to this tension. Thus, the extent to which it is possible for the national level, for example, to recognize potential, sometimes very specific, conflicts of justice within the urban context (here Glasgow) must be called into question. The British government’s plan to introduce low-emission zones across the board without taking specific local problems into account, despite the reference to the necessary local contextualization, seems to be a symptom of this. Consequently, any conflict between the governing levels in relation to the question of who the ‘translator’ is, which is evident in many places within the material, has a notable effect on individual components of the norm translation and, thus, also on the impact of justice-related claims.

Threats of legal intervention and the capacities of the individual cities under consideration provide a further perspective through which we can understand the translation of the EU Directive 2008/50. Both categories follow on from points already presented, even if, at this point, they are seen as representing a particular action that promotes or hinders justice within the cases analyzed. In the context of norm translation and with a view to the specific cases, legal intervention means considering a particular translation as inadequate and asserting this before a legal authority. There is undoubtedly a difference in whether such a claim is formulated out of personal concern or out of the function of an observer. Thus, the legal pressure to comply, as projected and received by the EU Commission, is seen, in all cases, as an external pressure factor that makes a specific action necessary and as a justification for a particular action. However, it remains unclear how and whether the local/regional contexts want to cooperate to develop the norm further. Although all the plans considered here refer to the fact that other political levels should work towards a revision of the EU directive itself, it remains vague as to which actors should participate and in which forums this should occur. The circular notion of norm translation thus remains largely unused in practice. At the local level, as is evident in the case of Hamburg, a passive behavior focusing on legal compliance seems to prevail.

However, this needs to be understood in light of a lack of material and immaterial resources present in all cases considered. In Hamburg and Glasgow, limited resources were used to justify the incapacity to translate Directive 2008/50 comprehensively. Brussels, to some extent, circumvented this by describing the air quality plan within a broad strategy, that is, as a strategic process rather than a ‘to do list’. What influence does this have on the translation of standards, especially concerning socioecological justice? One could say that such a translation should necessarily be oriented towards the design possibilities of the ‘target language’. However, more specifically, as many *environmentally* sound options as there are for translating the EU norm, specific measures can only ever work within the framework of local restrictions. Does this mean that the Brussels air quality plan is largely unusable compared to the plans of Hamburg and Glasgow, which set out concrete cost-benefit calculations? This question cannot be definitively answered. However, it seems plausible to suggest that the Brussels’ plan is more successful at detaching itself from the original ‘language’, in this case, of the EU Directive and the constraint of its literal implementation. A ‘work of its own’ is created in the context of a local transformation process, even if this is at the expense of recognition, understood as a legally correct transposition of the Directive and comparability with other translations (cf. Berger and Esguerra 2018, pp. 5 f.). To what extent this makes the Brussels air quality plan a ‘better’ translation, in the long run, remains to be answered here.

## Conclusion

This article has elaborated on the interplay between EU environmental policy, namely the EU AAQ Directive 2008/50, and the socioecologically ‘just’ transition of urban mobility. We have observed that an environmental justice lens, which constitutes an essential aspect of a successful sustainable transition in sectors such as the mobility sector, remains vastly omitted in EU policy studies. By conceiving of the political dynamics within the EU’s multilevel governance system as a process of norm translation, we have further accounted for the inherent agency of regional and local actors in putting complex and ambiguous justice norms ‘into use’ and, thus, investigated their central role in providing for an EU-wide sustainable transition. This contribution has advanced two main arguments, with regard to these research objectives.

First, our exploratory analysis underscored the notion that the implementation of justice-related norms that move through EU environmental legislation does not ‘just happen’. Conversely, the results of the exploratory qualitative-interpretative analysis of three cases of these norm dynamics—Brussels, Glasgow, and Hamburg—convincingly show a dynamic process of translation into urban policy evolves in different ways that affect diverse and potentially ambiguous justice dimensions. Our analysis identified the essential aspects that underscore the complexity *and* potential of local policy implementation processes. The concept of norm translation has been particularly helpful to disentangle these dynamics. Adapting this concept has allowed us to analytically distinguish between the diverging operationalization of meanings in AAQ governance. Making EU norms effective, in that regard, is a task for ‘just’ translation, that also needs a sharper reflection upon in EU policy and governance research.

Second, as ‘hard law’, EU environmental regulations put high pressure foremostly on regional and local, but also on national actors, legally complying with regulations such as Directive 2008/50 is, therefore, non-negotiable, and at times even opens a window for regulatory measures, such as driving bans. As our analysis has shown, this pressure may trigger ‘incomplete’ translations of the normative aims in question. Through a justice lens, sustainably transforming mobility (and energy) systems is an ambiguous endeavor. On the one hand, regulations such as the AAQ directive react against prevailing injustices, both locally and globally. On the other hand, every measure that aims to limit those injustices can be contested because of its potential to create new injustices, such as social or economic exclusion. Acknowledging and weighing these normative claims is a critical task within norm translation dynamics. It precludes integrating different forms of knowledge and requires political resilience and resources. While EU environmental policy might consequently be perceived as ‘fuel’ for decisive local action, it might at the same time intervene in more holistic and long-time efforts for sustainable mobility transitions. This finding contributes to the emerging debate within EU policy studies about the limits and future direction of implementation and compliance research (see, for instance, Heidbreder 2017). In particular it underscores the puzzling question of how to translate normative meanings under the condition of functional subsidiarity. While this paper suggests that the translation of norms in EU governance should be made more tangible in principle, we also conclude that the sociocultural dynamics of translation are most evident at the local level. While EU regulation may dictate *that *translation should occur, the *how *of that translation and the reception of regulation necessarily remain a contested instance.

Our contribution also has implications for the future development of EU governance and policy practice. Particularily, we derive the augmented importance for ‘trans-level’ exchange and capacity building beyond hierarchical steering from these two main results discussed above. Especially in the transport & mobility sector, many networks and instruments such as the *CIVITAS *network and the *Sustainable Urban Mobility Plan *(SUMP) framework are already providing this space (Werland 2020; Pflieger 2014). However, to sustain and evaluate their transformative capacities for a mobility transition, further institutional and procedural groundwork is needed. For example, providing cities and local authorities, more generally speaking, with the space and capacity to translate allows for a more holistic approach to normative aspirations that guide political regulations (Kern 2019; Bulkeley et al. 2018). This, from our point of view, requires a rethinking of the interactions within the multilevel governance system. A coherent and “robust” translation of EU norms requires interaction with and empowerment of the local level rather than relying on the linear diffusion of norms through the system of national law.

As with every piece of research, this study does have its limitations. To gain a more robust picture of the dynamics of norm translation in the EU multilevel system, further refinements of the theoretical and conceptual framework are required. Such an effort could improve the emancipatory potential of the approach for EU studies and advance the norm-theoretical framework itself. To that extent, our contribution must be understood as a conceptual ‘field trial’ rather than a comprehensive theoretical engagement.

Beyond that, in our explorative analysis of the translation dynamics of the EU Directive 2008/50, we have used a combination of IR norm-theoretical research with literature from environmental justice. Justice is predominantly referenced in norm research in the context of democratic-transitional processes (Zimmermann 2017a; Berger 2017). While this literature has also been central to developing the argument of this contribution, we see merit in further examining the stimulating nature of norm research and justice in a socioecological understanding. Our analysis has pointed to interesting dynamics regarding identity formation and recognition-based claim-making in sustainable transitions. Nevertheless, this interplay, which is moderated through norms and their translation, clearly offers opportunities for further elaboration.

Furthermore, our empirical work is, of course, limited to a comparably narrow range of sample cases. The study of three urban contexts allowed us to engage thoroughly with the material and provided exciting results. Nonetheless, we are well aware that a larger, comparatively orientated study of translation contexts, also involving, for instance, Eastern European contexts, would possibly add further validity to our argument. Finally, contextualizing the results of our analysis within other policy dynamics affecting the nexus of mobility and energy policy would further illuminate the horizontal relationships between different pathways for EU norms. Whereas the mobility sector is the single most important sector implementing the AAQ Directive, the EU norm is also relevant for other sectors. For example, it is likely that similar justice effects also occur in the housing sector (Jenkins et al. 2016). However, in this contribution we were unable to accommodate this perspective. Furthermore, regarding the identity-related contents of norm translation, we detected an incapacity for established political science methods to access the process of cognitive identity-building.

With our contribution, we seek to inspire a critical debate on the trajectories of EU policies at the nexus of different fields such as environment, mobility, and energy. Our results show that the translation of norms at this intersection triggers conflicts of justice that require political will and resilience to address. We believe this debate will gain further momentum at a time when the *EU Green Deal* has set out to provide a reconfiguration of the EU’s normative fundament (Fernandez et al. 2021). As we have demonstrated, making this aspiration a reality is arguably a task that involves much more than ‘just’ translation.

## Appendix

﻿

**Table 5:** Details of the documents analyzed

Case	Documents considered for the content analysis (date; cited as)
Hamburg (original documents in German)	Clean Air Plan, second update (2017; LRP FHH) Documentation of objections to the draft of the Clean Air Plan (2017; objections LRP FHH) Minutes of the joint public meetings and hearings of the Environment and Transport Committee of the Hamburg Parliament of 12.09. and 04.10.2017 (Committee minutes 12.09.17; Committee minutes 04.10.17). Report of the Environment Committee of the Hamburg Parliament on the Implementation of the Clean Air Plan (2017; Report UA)
Brussels (original documents in French)	Regional Air—Climate-Energy Plan (2016; LRP RBC) Emergency Plan for Pollution Peaks (2017; PUPP) Context Report on the LRP RBC (2015; Context RBC) Documentation of citizen participation in the RBC LRP (2016; RBC participation)
Glasgow	Framework Clean Air Plan for Great Britain (2017; LRP UK) Clean Air Plan for the Glasgow Air Quality Management Area (2015; LRP GLA). Guidelines for the design of local LRPs (2016; PG LRP) Cleaner Air for Scotland Strategy (2015; CAFS) Documentation of the public participation on the LRP UK (2017; Consultation A) Documentation of public participation on compensation mechanisms (2018; Consultation B) Documentation of public participation on Low Emission Zones (2017; LEZ-C)

## Footnotes

1 In the context of this contribution, *AAQ* refers to the quality of air as measured at a specific point and time. The AAQ therefore differs from place to place and is, therefore, by definition, a subject to be understood in local terms. This limitation implies that *air pollution* refers to the deterioration of AAQ by pollutants whose concentration can be measured accordingly, and which impact the ‘ambient environment’. These pollutants, such as ozone (Oz), nitrogen oxides (NO2; NOx), and particulate matter (PM), differ from what is commonly understood as *Greenhouse Gases* (GHG) which are measured (and have effects) on a more global scale. Finally, the *emission* of these pollutants refers to the output into the air generated through technical processes, such as combustion, abrasion, and other chemical reactions; see World Health Organization (2017).

2 While reacting to the foremost local problems occurring in the ambient air, the AAQ Directive needs to be also understood, in the broader context of low-carbon mobility, as a means to react to the climate crisis (Mattioli 2016; Bollen et al. 2010). In doing so, cities become real-world ‘laboratories’ to test new mobility solutions and foster sustainable transitions of mobility and energy (Kern 2019). This adds another layer to the debate that has recently gained traction under the concept of “climate justice” (Bulkeley et al. 2014; Schlosberg and Collins 2014).

3 A factor that is also highlighted in the global health regime, as for instance, by the World Health Organization (2017).

4 These protests seem to go hand in hand with resistance to other post-modern transformations such as gender equality, multiculturalism, digitalization, and more of similar reach. In this context the normative aim of ‘clean air’ does not seem to be the only vantage point for critique but provides an opportunistic structure for a general presentation of non-acceptance; see for example Daggett (2018).

5 We do acknowledge that this research did not just emerge from one day to the other. Sidelined and deemed “dissident” (Manners and Whitman 2016) by mainstream EU studies, alternative concepts of EU policymaking have been nurtured in other disciplinary debates such as international political sociology (Adler-Nissen and Kropp 2015) and environmental studies (Wagenaar 2015; Hajer 2003). Therefore, it is no coincidence that concepts of these disciplines and fields have recently been imported into EU studies, which has consequently undergone a vibrant and multi-faceted re-conceptualisation through self-reflection of its normative premises (Mc Mahon 2018).

6 This reflects the critical foundations of environmental justice scholarship in the US discourse on racial inequalities, that was later expanded to account for the different dimensions of the ‘critical trias’ of gender, class, and race (Cutter 1995) as well as the intersectional relationship between these dimensions (Malin und Ryder 2018).

7 For practical reasons, we only considered documents in German, English, and French (in specific individual cases) which means that we excluded certain documents (namely those in Flemish and Dutch) related to Brussels. A list of all documents analyzed is provided in Table 5 in the appendix to this article.

8 Arrêté du Gouvernement de la Région de Bruxelles-Capitale portant modification de divers arrêtés relatifs à la qualité de l’air ambiant (2011) for Belgium/Brussels Region; Neununddreißigste Verordnung zur Durchführung des Bundes-Immissionsschutzgesetzes (2010) for Germany; The Air Quality Standards (Scotland) Regulations (2010) for the United Kingdom/Scotland.
